# Supplementary material for: A novel P2X2‐dependent purinergic mechanism of enteric gliosis in intestinal inflammation
Source: EMBO Mol Med. 2020 Dec 17;13(1):e12724. doi: 10.15252/emmm.202012724 (PMC7799361; doi:10.15252/emmm.202012724)
Supplement: Supplementary file 1 — Appendix [file EMMM-13-e12724-s001.pdf]

Title:

A novel P2X2-dependent purinergic mechanism of enteric gliosis in intestinal inflammation

Short Title: **P2X2 signaling in enteric glia**

Authors:

Schneider R.[1], Leven P.[1], Glowka T.[1], Kuzmanov I.[1], Lysson M.[1], Schneiker B.[1], Miesen A.[1], Baqi Y.[4], Spanier C.[2], Grants I.[3], Mazzotta E.[3], Villalobos-Hernandez E.[3], Kalff J.C.[1], Müller C.E.[2], Christofi F.L.[3], Wehner S.[1]

Table of contents:

Appendix Fig S1-5 + Fig legends

Appendix Table S1-4

Appendix Figure S1

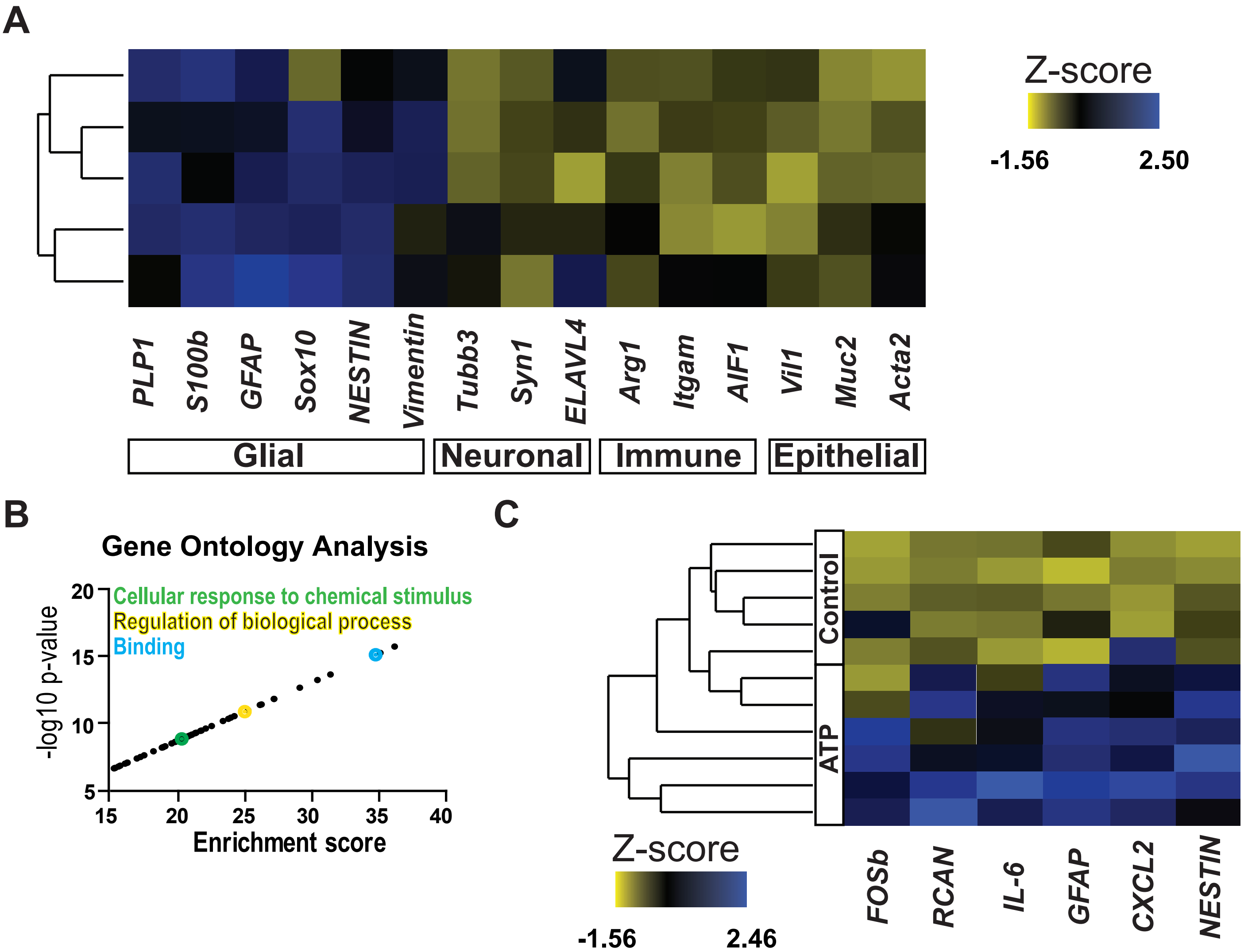

Appendix Fig S1: ExATP induces gliosis in enteric glia cells.

A: Heat maps of indicated genes in ATP-treated msEGCs compared to control. **B:** Visual representation of GO terms associated with enriched genes in ATP-treated msEGCs compared to control. **C:** Heat maps of indicated genes in ATP-treated msEGCs compared to control (n=5 for untreated EGCs and n=6 for ATP-treated EGCs).

Appendix Figure S2

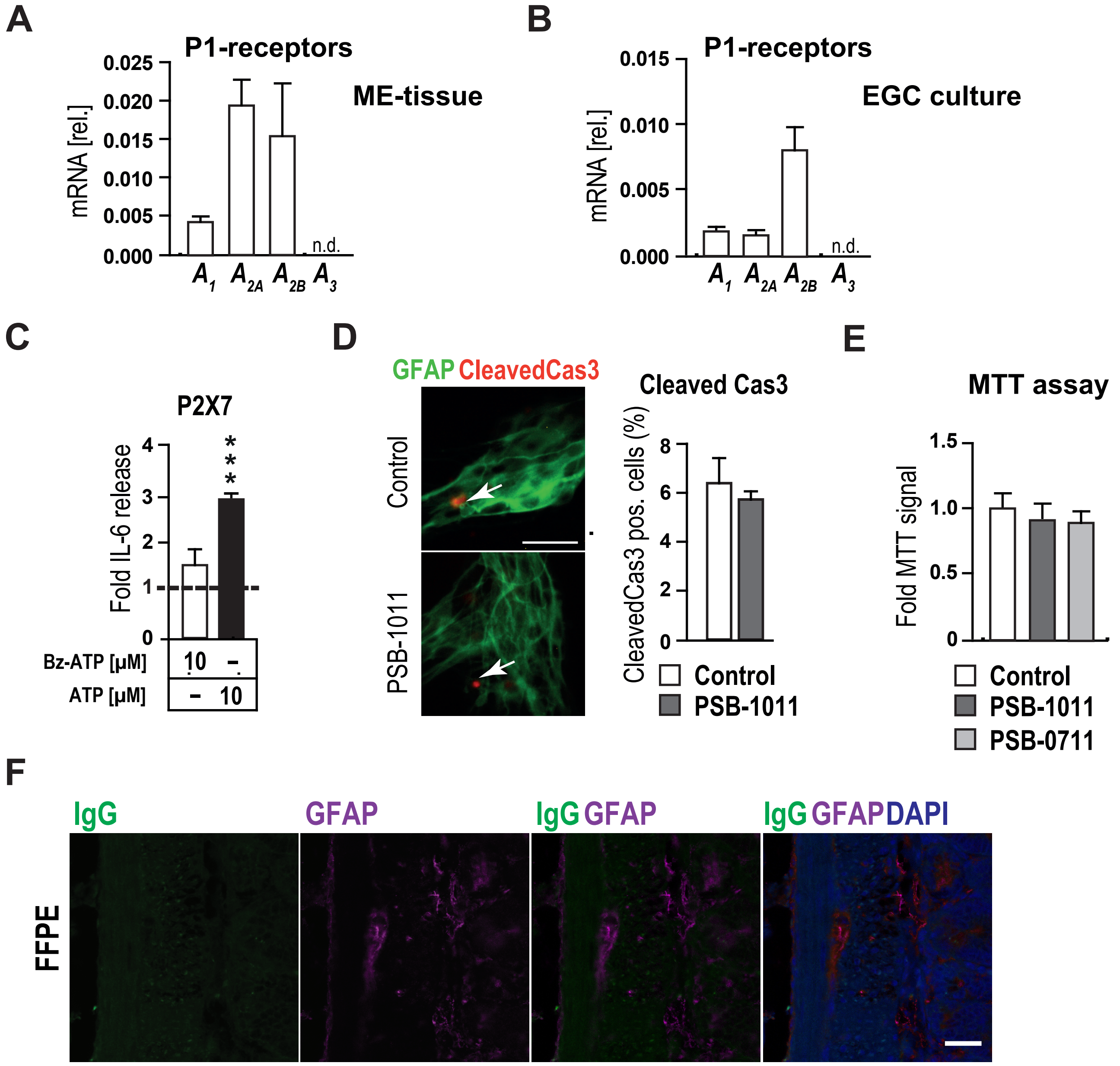

Appendix Fig S2: ATP-induced gliosis is mediated by p38-MAPK and P2X2-purinergic signaling

A+B: qPCR analysis of P1-purinergic receptors in msEGCs isolated from ME (n=3) or from cultured cells (n=6), respectively. **C:** Protein release measurement by ELISA of IL-6 in EGCs. Cells were treated with the P2X7 agonist bz-ATP (10µM) or with ATP (10µM) for 24h. **D:** Cleaved caspase 3 analysis in EGCs. Representative fluorescence images show GFAP (green) positive EGCs and cleaved caspase 3 (CleavedCas3, red) positive cells. Cells were treated with P2X2-antagonist PSB-1011 (20µM) for 24h. Scale bar 50µm. **E:** MTT assay analysis in EGCs after treatment with PSB-1011 and PSB-0711 (both, 20µM) for 24h. **F:** Representative confocal images show GFAP (violet) positive EGCs and rbIgG control (green). Scale bar 50µm.

Data information: In **A+B** data are represented as mean +SEM normalized to *GAPDH* expression. In **C** data are represented as fold-change +SEM; n=8. In **D** data are represented as percentage +SEM of total cell numbers; n=5. Statistics were done in **C** by applying Student´s-t-test. * indicates significance to control**.**

Appendix Figure S3

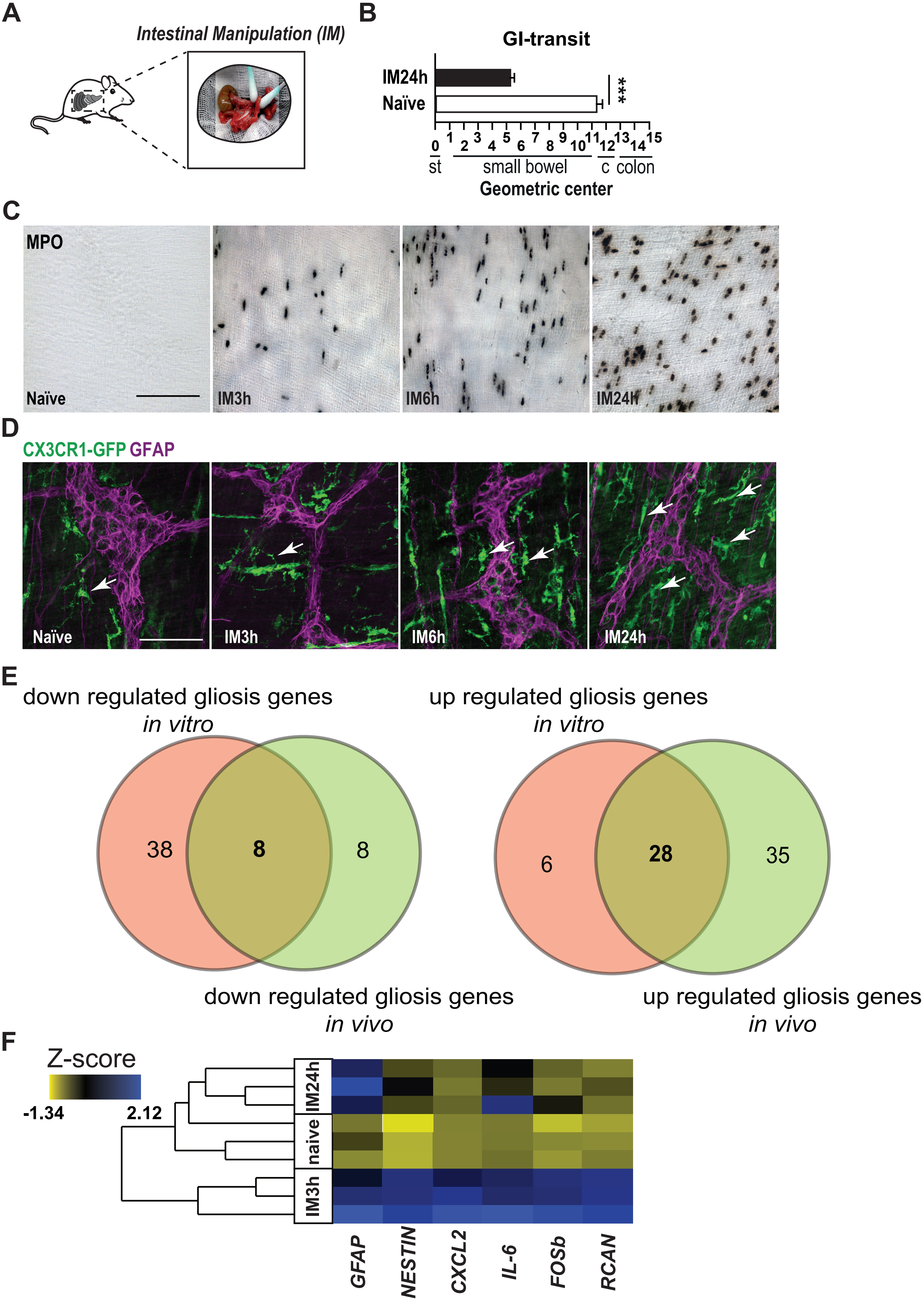

Appendix Fig S3: Intestinal inflammation induces enteric gliosis

A: Schematic overview of the animal model of postoperative ileus (POI). **B:** Gastrointestinal transit measurement in naïve and POI mice after IM24h. Data are represented as mean +SEM; n=7. **C:** Representative bright field images of MPO positive cells in the course of POI. Scale bar 50µm. **D:** Representative confocal images of GFAP-positive EGCs (violet) and CX3CR1-positive macrophages (green) at different time points after surgery. White arrows mark resident and later activated macrophages. Scale bar 25µm. **E:** Venn diagram of up and down regulated gliosis genes expressed in EGCs *in vitro* and *in vivo*. **F:** Heat map of all significantly changed genes from POI mice at different disease stages and naïve mice; n=3 for each group.

Data information: In **B** data are represented as mean +SEM; n=7. Statistics were done in **B** by applying Student´s-t-test. * indicates significance to naïve animals**.**

Appendix Figure S4

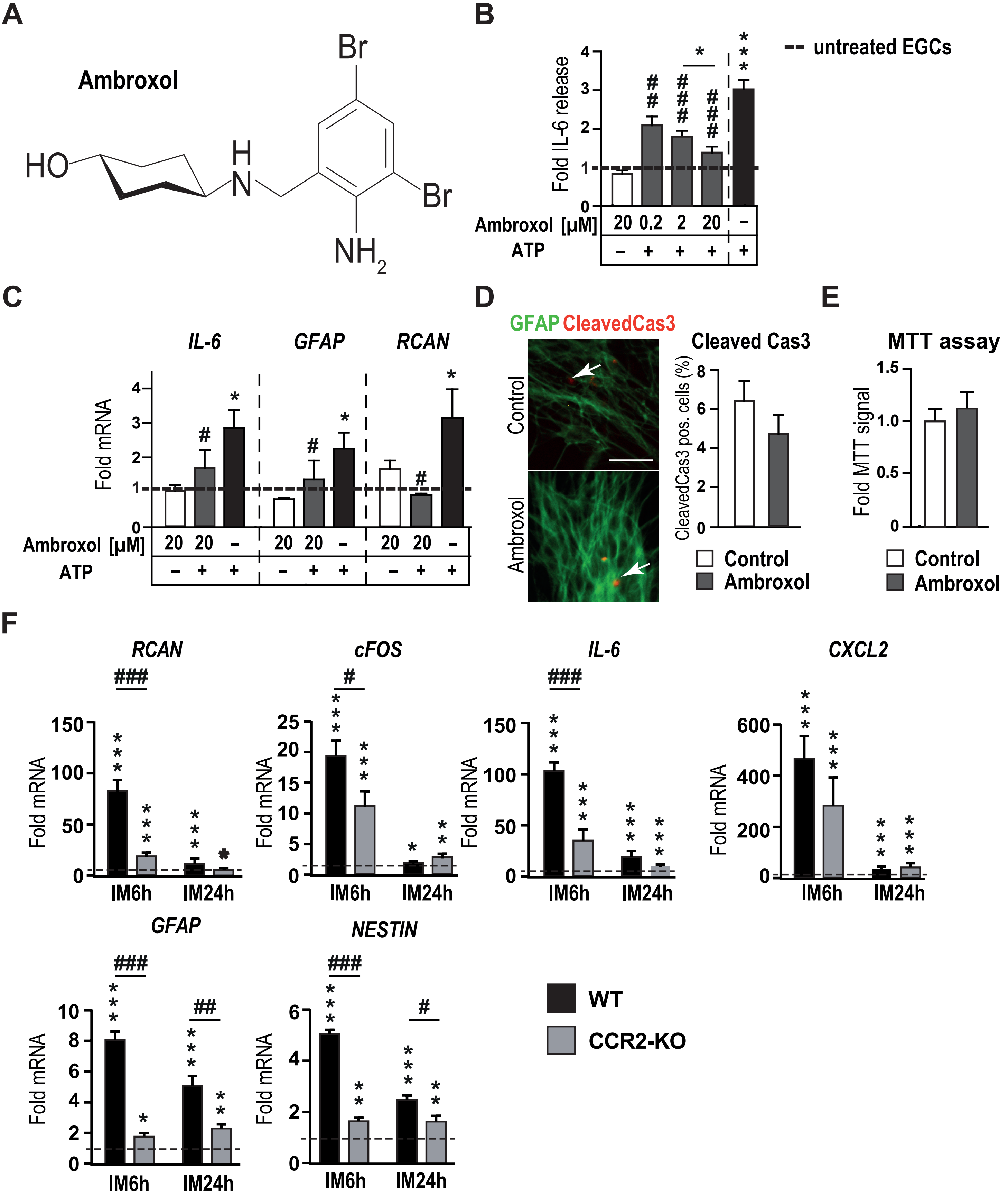

Appendix Fig S4: P2X2 signaling inhibition by ambroxol improves clinical symptoms in POI

A: Chemical structure of ambroxol. **B:** Ambroxol blocks ATP induced IL-6 release in msEGCs; ELISA measurement of IL-6 in msEGCs upon treatment with ambroxol alone or together with ATP (10µM) for 24h (n=8-16). **C:** Gene expression analysis by qPCR of *IL-6, GFAP and RCAN* in EGCs. Cells were treated with ambroxol (20µM) alone or together with ATP (10µM) for 6h; n=6. **D:** Cleaved caspase 3 analysis in EGCs. Representative fluorescence images show GFAP (green) positive EGCs and cleaved caspase 3 (CleavedCas3, red) positive cells. Cells were treated with ambroxol (20µM) for 24h. Scale bar 50µm. Data are represented as percentage +SEM of total cell numbers; n=5. **E:** MTT assay analysis in EGCs after treatment with ambroxol (20µM) for 24h. **F:** qPCR analysis of indicated gliosis marker in wt and $CCR2^{-/-}$ mice that underwent IM (n=7).

Data information: In **B**, **C** and **F** data are represented as fold change +SEM. Statistics were done in **B**,**C** and **F** by applying Student´s-t-test and one-way ANOVA with a subsequent Bonferroni test. * indicates significance to control**, #** indicates significance to ATP treatment or between wt and $CCR2^{-/-}$ mice.

Appendix Figure S5

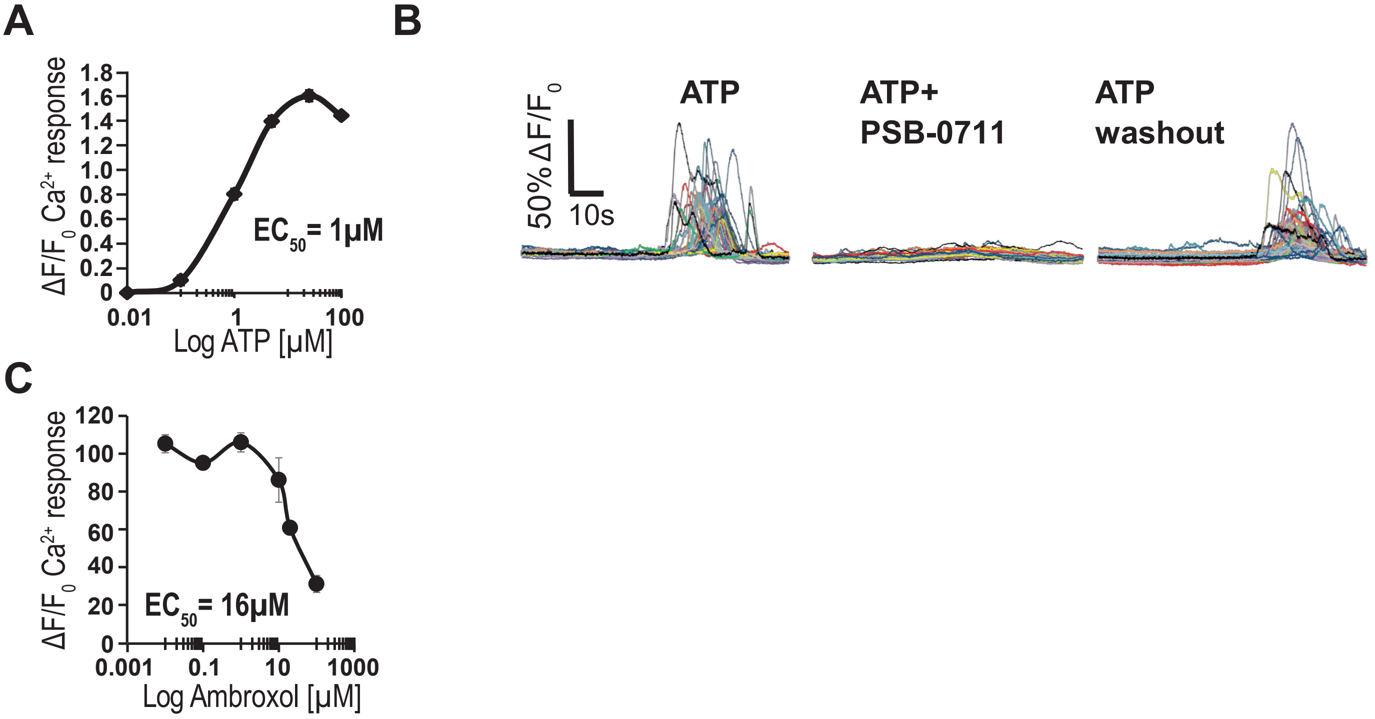

Appendix Fig S5: ExATP induces gliosis in human enteric glia

A: Representative concentration-response curve of ATP to induce an increase in intracellular free Ca^{2+} levels in human HEK-P2X2 cells. The EC_{50}=1µM (cells tested at increasing concentrations of ATP ranging from 0.1µM to 100µM applied at 15 min intervals, see results); n=88. **B:** Representative experiment showing reversible effect of PSB-0711 on ATP-induced Ca^{2+} responses in sniffer cells. **C**: Ambroxol caused a concentration-dependent inhibition of the ATP triggered calcium response. The ambroxol EC_{50} is 16µM; n=495 cells.

Appendix Table S1: Human subjects and GI surgical specimens for IHC and hEGC cultures

#	Patient ID	Sex	Age	Surgical procedure	GI region
**1**	PSG283	female	35	Colectomy partial open	*descending colon*
**2**	PSG285	female	65	Colectomy total with ileostomy	*descending colon*
**3**	PSG286	female	36	Sigmoidectomy	*descending colon*
**4**	PSG288	male	68	Colectomy with partial coloproctostomy	*descending colon*
**5**	PSG291	female	56	Colectomy partial w/coloproctostomy laparoscopic	*descending colon*
**6**	PSG294	female	66	Colectomy partial w/coloproctostomy	*descending colon*
**7**	hPOI-001	male	55	Colectomy partial w/coloproctostomy laparoscopic	*descending colon*
**8**	PSG287	female	52	Colectomy partial open	*ascending colon*
**9**	PSG072	male	64	Colectomy partial w/colostomy	*ascending colon*
**10**	PSG077	female	57	Colectomy partial laparoscopic	*descending colon*
**11**	PSG154	male	64	Colectomy partial w/ colostomy	*descending colon*
**12**	PSG270	male	64	Colectomy partial w/coloproctostomy open	*descending colon*
**13**	hPOI-010	male	52	Colectomy partial w/coloproctostomy lap.	*descending colon*
**14**	PSG289	female	50	Roux-en-Y open	*jejunum*

Appendix Table S1 summarizes information on the 14 human subjects that gave written consent, to procure gut surgical tissue for *in vitro* studies. Tissue was used to isolate ganglia, for preparing hEGC cultures for study, or paraffin sections for immunofluorescent staining (including hEGC/IF, colon P2X2-ir and qPCR analysis). Studies were conducted under IRB protocols.

Appendix Table S2: Human subjects and GI surgical specimens for analysis of gliosis

#	Patient ID	Sex	Age	Start Time of procedure	Time points of sample collection
**1**	160303_hME	male	55	*09:18*	*11:35 and 14:00*
**2**	160331_hME	male	85	*08:50*	*10:50 and 12:16*
**3**	160405_hME	male	79	*09:14*	*11:15 and 12:40*
**4**	180220_hME	male	80	*8:30*	*12:20 and 13:10*
**5**	180221_hME	male	74	*10:00*	*13:45 and 14:15*
**6**	180424 hME	female	61	*08:30*	*11:35 and 13:50*
**7**	180821_hME	male	59	*9:00*	*12:15 and 13:35*
**8**	181029_hME	female	73	*07:45*	*10:30 and 11:15*
**9**	190429_hME	female	61	*9:15*	*13:58 and 15:13*
**10**	190506_hME	male	76	*8:55*	*12:12 and 13:00*
**11**	190805_hME	male	29	*10:24*	*12:00 and 14:29*
**12**	190806_hME	female	30	*10:01*	*12:25 and 13:30*
**13**	190808_hME	female	70	*10:00*	*13:40 and 18:20*
**14**	190917_hME	female	51	*9:21*	*12:07 and 12:55*
**15**	191017_hME	male	75	*9:11*	*13:38 and 15:22*
**16**	191023_hME	male	57	*9:11*	*11:20 and 14:10*

Appendix Table S2: Information on 16 patients who underwent pylorus-preserving pancreaticoduodectomy and gave written consent to procure jejunal tissue for qPCR studies. The tissue was used for RNA isolation, cDNA preparation and gene expression analysis. The study was conducted under protocols permitted by the ethical committee of North Rhine-Westphalia, Germany.

AppendixTable S3: Oligonucleotide primers

Gene	Forward	Reverse
mP2X1	GGATGGTGCTGGTACGAAACA	CACTGACACACTGCTGATAAGG
mP2X2	TTTACTTCGTGTGGTACGTCTTC	ACATGGTGATCCCCTTGACTT
mP2X3	AAAGCTGGACCATTGGGATCA	CGTGTCCCGCACTTGGTAG
mP2X4	ACCAGGAAACGGACTCTGTG	TCACGGTGACGATCATGTTGG
mP2X5	AGAAGGTGGGGCTGCTCTA	ACAGCACTCTGCAGGGAAGT
mP2X6	GTAGTCTACGTGATAGGGTGGG	CACGAGGAAGTTGGTTACCAG
mP2X7	GACAAACAAAGTCACCCGGAT	CGCTCACCAAAGCAAAGCTAAT
mP2Y1	TGTGAAGGCACGAGATCCTA	AGGTGGCACACACTGGTCTT
mP2Y2	AACTTTGTTTCCTCTAGAGCGTG	CAGGAAGAACAGGTTCCCAG
mP2Y4	AGCAGAACACCATTGACTGC	CAAGGAGTCTGCACTGGTCA
mP2Y6	GCTTGCTAAAGGGGCTTTCT	TACTGTTGCTGCCCATCTTG
mP2Y10	TGACAAGGAGTAGTGGATGCC	ATTGCAATTGCTCTCAGCAC
mP2Y12	GGACACTTTCCCGTATCCAG	CAGGTCGGGTAGCACGTTAT
mP2Y13	GTGGGTTGAGCTAGTAACTGCC	CAGCTGTGTCATCCGAGTGT
mP2Y14	AGCCAGACGTGAAGGAGTTC	AAGGCAAGCTTCGTCAACAG
mA1	GATCGGTACCTCCGAGTCAAGA	GGCCTACCACAAGGGAGAGAA
mA2a	TCCTCACGCAGAGTTCCATCT	GAATGACAGCACCCAGCAAA
mA2b	TCTTCCTCGCCTGCTTCGT	CTCGCTCGTGTCCCAGTGA
mA3a	CTTCTATGCCTGCCTTTTCATGT	ATTCTTCTTTGAGTGGTAACC
mGFAP	ACATCGAGATCGCCACCTAC	CCTTCTGACACGGATTTGGT
mNESTIN	AGATCGCTCAGATCCTGGAA	AGGTGTCTGCAAGCGAGAGT
mRCAN	CTTGTGTGGCAAACGATGATG	TGGTGTCCTTGTCATATGTTCTG
mFOSb	ATGGGCTCTCCTGTCAACAC	ACGGAGGAGACCAGAGTGG
mCCL2	Taqman Assay: Mm00441242	
mIL-6	Taqman Assay: Mm00446190	
mCXCL2	TCCAGGTCAGTTAGCCTTGC	CGGTCAAAAAGTTTGCCTTG
mCD73	GCCAAGATAGAGGTGAAACCA	AGCTGCCCCTTATGGAAGAT
mENTPD1	GCCAAGATAGAGGTGAAACCA	AGCTGCCCCTTATGGAAGAT
mENTPD2	Taqman Assay: Mm00515450_m1	
mENTPD3	CGGCCACTGATACACGTAGA	TTACACTCATCCAGATCCGC
mENTPD8	AGGACACAGGAGTGGTCAGC	CAGCTCTTCAGGCTTTCCC
18S-RNA	GTGGAGCGATTTGTCTGGTT	ACGCTGAGCCAGTCAGTGTA
hGFAP	CTGCGGCTCGATCAACTCA	TCCAGCGACTCAATCTTCCTC
hNESTIN	GGGAGTTCTCAGCCTCCAG	GGAGAAACAGGGCCTACAGA
hRCAN	GCTCCGCCAAATCCAGACAA	GCTGCGTGCAATTCATACTTTTC
hFOSb	CAA GCG GAG ACA GAC CAA CT	AGT CAG ATC AAG GGA AGC CA
hIL-6	GTCAGGGGTGGTTATTGCAT	AGTGAGGAACAAGCCAGAGC
hCXCL2	GGCAGAAAGCTTGTCTCAACCC	CTCCTTCAGGAACAGCCACCAA

Appendix Table S3 summarizes information on all used qPCR primers.

Appendix Table S4: Oligonucleotide primers

Target	Dye/Dilution	Secondary antibody*	Use	Company
MHCII	PE / 1:200		FACS	Biolegend Clone:M5/114.15.2 #107607
CD45	Pacific blue / 1:200		FACS	Biolegend Clone: 30-F11 #103126
Ly6C	APC / 1:300		FACS	eBioscience Clone: HK1.4 # 17-5932-82
GFP	1:600	AlexaFluor488	IHC	Novus biologicals Clone: 1A8 #NB100-1614
MHCII	1:400	AlexaFluor647 or Cy3	IHC	BioLegend Clone:M5/114.15.2 # 107602
GFAP	1:1000	AlexaFluor647	IHC/ICC	Synaptic System # 173002
Sox10	1:400	Cy3	IHC/ICC	Santa Cruz # sc- 17342
Ki67	1:400	Cy2	IHC	Abcam Clone: SP6 # ab16667
P2X2	1:400	Cy2	IHC, WB	Alomone # APR-003
αSMA	1:1000	Cy2	ICC	Dianova # APR-003
FOSb	1:200	Cy3	IHC	Santa Cruz # sc52
s100β	1:1000	AlexaFluor647	ICC	Synaptic System # 287004
Cleaved-Caspase 3	1:400	AlexaFluor647	ICC	cell signaling # cs9664
P-p38 MAPK	1:400/ 1:1000	Cy2	ICC,WB	cell signaling # cs9212
p38 MAPK	1:1000		WB	cell signaling # cs4631

Actin	1:5000		WB	SIGMA #A5316
s100β	1:500	Cy3	IHC/ICC	Abcam # ab11178
αSMA	1:500	Cy2	ICC	Abcam # ab18147
antifibroblast / epithelial cell	1:500	Cy2		Novus biologicals # NB600–777

Appendix Table S4 summarizes information on all used antibodies.
