# Supplementary material for: A novel P2X2‐dependent purinergic mechanism of enteric gliosis in intestinal inflammation
Source: EMBO Mol Med. 2020 Dec 17;13(1):e12724. doi: 10.15252/emmm.202012724 (PMC7799361; doi:10.15252/emmm.202012724)
Supplement: Supplementary file 2 — Expanded View Figures PDF [file EMMM-13-e12724-s002.pdf]

Expanded View Figures

Figure EV1. ExATP induces gliosis in enteric glia cells.

A Histological analysis of EGC culture purity by quantification of EGCs and fibroblasts *in vitro*. Representative immunofluorescence image shows GFAP (violet)-positive EGCs and α smooth muscle actin (αSMA, green)-positive fibroblasts with DAPI as counterstain. Scale bar 50 μm.
B Representative immunofluorescence image of s100β (violet)- and Sox10 (green)-positive EGCs with DAPI as counterstain. Scale bar 10 μm.
C PCA plot of gene expression by ATP-treated and untreated EGCs. Blue circles represent ATP-treated EGC cultures, and white circles are matching controls; n = 5–6, respectively.
D Heat map of ATP-target genes, showing a collection of known target genes of ATP signaling (n = 5–6, msEGCs).
E Representative Western blots of phospho-p38-MAPK (pp38) and p38-MAPK (p38) in 1 h ATP-treated EGCs. Actin was used as loading control (n = 3, msEGCs).
F Representative images of GFAP (violet)- and phospho-p38-MAPK (pp38, green)-positive msEGCs with or without ATP treatment (100 μM) for 1 h. White arrows show pp38-positive (ATP-treated) or negative (untreated) EGCs. Scale bar is 10 μm.
G Effect of p38 inhibition on ATP-induced IL-6 release. Cells were treated with the p38-MAPK inhibitor SB203580 (1, 5, 10 μM) alone or together with ATP (100 μM) for 24 h. ELISA measurement of IL-6 in msEGCs supernatants (n = 7–22, msEGCs).
H Effect of p38 inhibition on ATP induced mRNA expression of gliosis markers in msEGCs. Cells were treated with SB203580 (10 μM) alone or together with ATP (100 μM) for 6 h (n = 4, msEGCs).

Data information: In (A), data are represented as percentage + SEM normalized to the total cell numbers, n = 8, msEGCs. In (G and H), data are represented as fold induction + SEM. Statistics were done in (G and H) by applying unpaired Student's t-test and one-way ANOVA with a subsequent Bonferroni test. * indicates significance to control, and # indicates significance to the ATP treatment with $*/^{\#}P < 0.05$, $^{\#\#}P < 0.01$, and $***/^{\#\#\#}P < 0.001$.
Source data are available online for this figure.

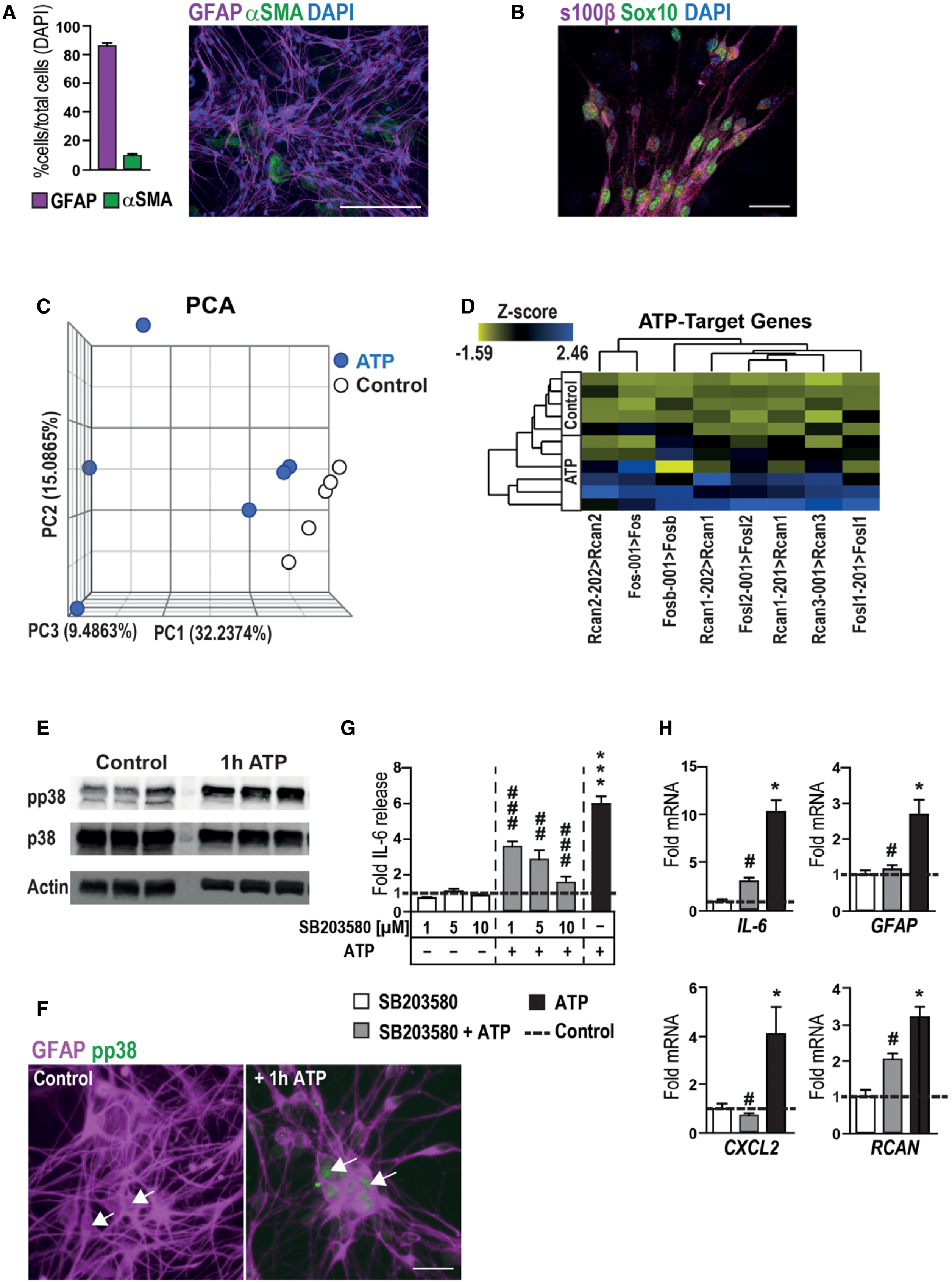

Figure EV1.

 © 2021 The Authors

Figure EV2. ATP-induced gliosis is mediated by p38-MAPK and P2X2-purinergic signaling.

A IL-6 release measurement by ELISA of IL-6 in msEGCs. Cells were treated adenosine (1 and 100 μM) or with ATP (100 μM) for 24 h; n = 14–16, msEGCs.

B Protein release measurement by ELISA of IL-6 in msEGCs. Cells were treated with P2 antagonist PPADS (5, 30 μM) alone or together with ATP (10 or 100 μM) for 24 h; n = 11–12, msEGCs.

C Protein release measurement by ELISA of IL-6 in msEGCs. Cells were treated with P2X2 antagonist PSB-1011 (0.2, 2, 20 μM) or PSB-0711 (0.2, 2, 20 μM) alone or together with ATP (10 μM) for 24 h; n = 9–13, msEGCs.

D Schematic overview of the isolation of msEGCs from small bowel muscularis externa of $GFAP^{cre}$-$Ai14^{fl/wt}$ mice: FACS-sorted $tdTomato^{+}$ msEGCs were either analyzed directly (*ME-tissue*) or in cultured msEGCs before tdTomato-FACS-sorting and further analysis; n = 3–6.

E Gene expression analysis by qPCR of *GFAP* and *Sox10* in msEGC cultures (n = 10) and mouse ME tissue (n = 10).

F, G Representative images of co-localization of GFAP (green) and $tdTomato^{+}$ msEGC (red) in the ME and in cultured EGCs. Scale bars 50 μm.

H–K qPCR analysis of P2-purinergic receptors in msEGCs isolated from ME (H, J; n = 3) or from cultured cells (I, K; n = 6), respectively.

L, M Representative Western blots of P2X2 in msEGCs transfected with siRNA-control or siRNA-P2X2 for 72 h together with an optical density measurement, see in M. Actin was used as loading control and normalization (n = 6, msEGCs).

Data information: In (A–C and E), data are represented as fold induction + SEM. In (H–K), data are represented as mean + SEM normalized to *GAPDH* expression. In (M), data are represented as optical density + SEM normalized to actin expression. Statistics were done by applying unpaired Student's t-test in (A-C, M and E) or both by unpaired Student's t-test and one-way ANOVA with a subsequent Bonferroni test in (B and C). * indicates significance to control, and # indicates significance to the ATP treatment with $^{\#}P < 0.05$, $^{**/\#\#}P < 0.01$, and $^{***/\#\#\#}P < 0.001$.

Source data are available online for this figure.

© 2021 The Authors

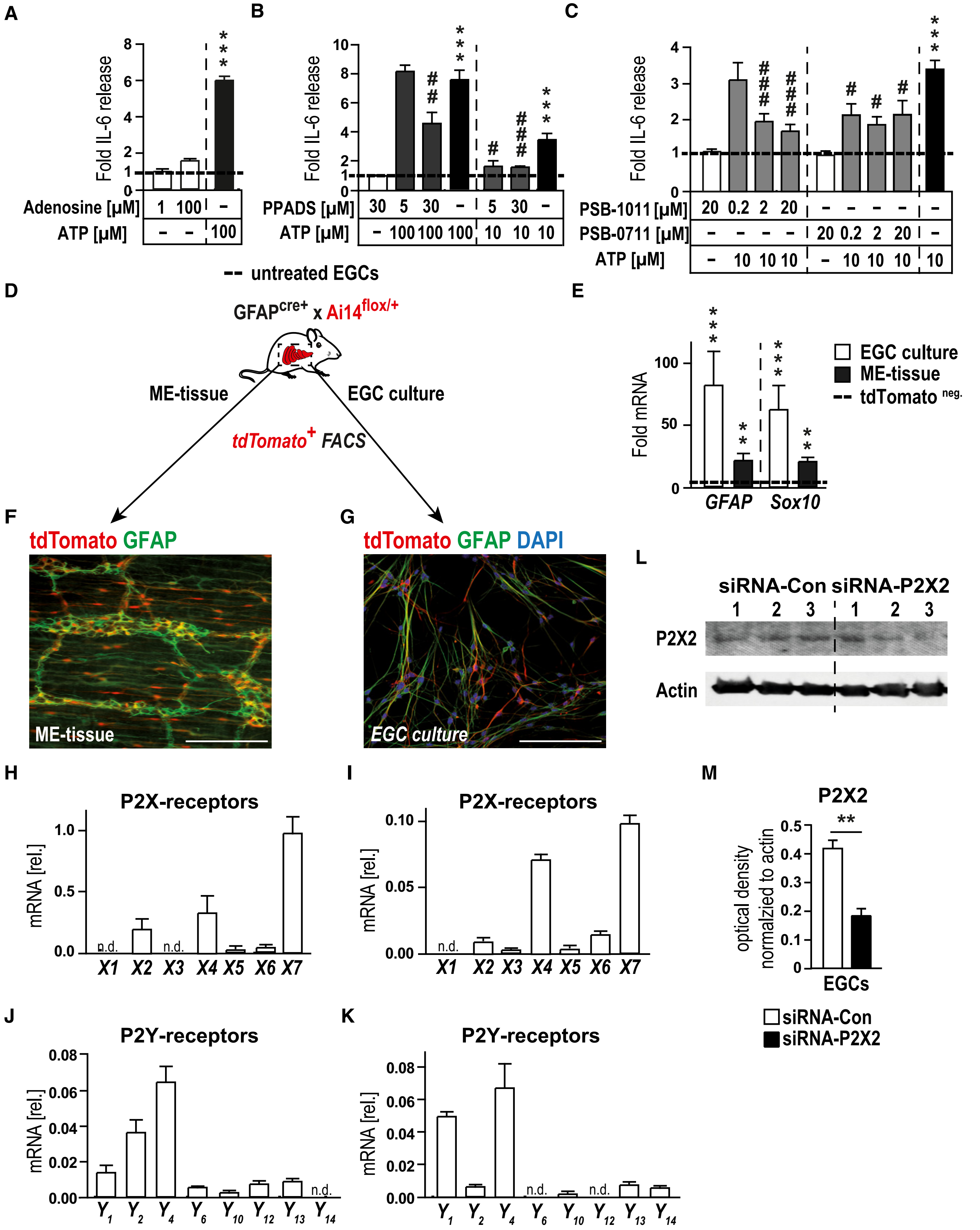

Figure EV2.

© 2021 The Authors

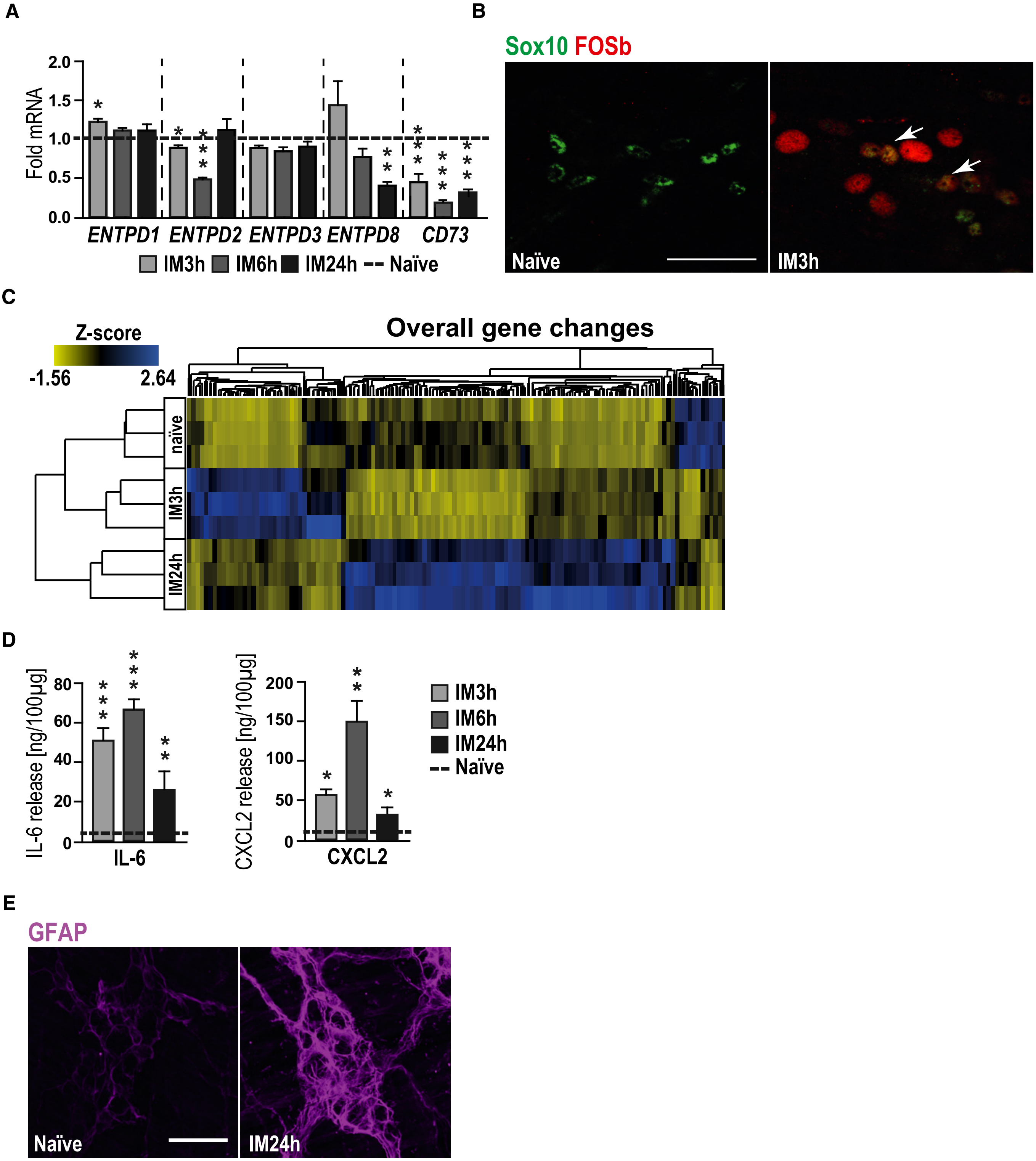
A
2.0
1.5
1.0
0.5
0.0
Fold mRNA
ENTPD1 ENTPD2 ENTPD3 ENTPD8 CD73
IM3h IM6h IM24h Naïve
B
Sox10 FOSb
Naïve
IM3h
C
Z-score
-1.56
2.64
Overall gene changes
naïve
IM3h
IM24h
D
IL-6 release [ng/100µg]
80
60
40
20
0
IL-6
CXCL2 release [ng/100µg]
200
150
100
50
0
CXCL2
IM3h
IM6h
IM24h
Naïve
E
GFAP
Naïve
IM24h

Figure EV3.

Figure EV3. Intestinal inflammation induces enteric gliosis.

A Gene expression of ectonucleotidases in POI mice at indicated disease stages.
B Representative confocal images of the activation marker FOSb (red)- and Sox10 (green)-positive EGCs (white arrows) in ganglia of intestinally manipulated mice (3 h after IM) or naïve mice. Scale bar 50 μm.
C Heat map of all significantly changed genes from POI mice at different disease stages and naïve mice; $n = 3$ for each group.
D Protein release analysis by ELISA of IL-6 or CXCL2 in POI mice at indicated disease stages.
E Representative confocal images of gliosis marker GFAP (violet)-positive EGCs in ganglia of intestinally manipulated mice (24 h after IM) or naïve mice. Scale bar 50 μm.

Data information: In (A), data are represented as fold change + SEM; $n = 7$, POI mice. In (D), data are represented as IL-6/CXCL2 protein in 100 μg tissue + SEM; $n = 7$, POI mice. Statistics were done in (A, D) by applying unpaired Student's *t*-test. * indicates significance to control with *$P < 0.05$, **$P < 0.01$, and ***$P < 0.001$.
Source data are available online for this figure.

Figure EV4. P2X2 signaling inhibition by ambroxol improves clinical symptoms in POI.

A Concentration-dependent inhibition of ATP-induced calcium influx in 1321N1-astrocytoma cells with recombinant expression of the human P2X2 receptor. ATP was used in a concentration corresponding to its EC80 value (1 μM). An IC_{50} value for ambroxol of 5.69 ± 1.06 μM ± SEM was determined. Inhibitory potency of ambroxol at P2X receptor subtypes X1-X7. At an initial test concentration of 20 μM, only the P2X2 receptor subtype was blocked by more than 50% indicating P2X2 receptor selectivity and no significant receptor inhibition was detected (n.d.) with other P2 receptor subtypes ($n = 6$).
B Protein release analysis by ELISA of IL-6 and CXCL2 in POI mice treated with ambroxol or vehicle at 24 h.
C Gene expression analysis by qPCR of *CCL2* and *TNFα* in POI mice treated with ambroxol or vehicle at IM3h and 24h; $n = 6$ POI mice.
D Representative FACS gating strategy of infiltrating cells in the ME of mice treated with ambroxol or vehicle. CD45, Ly6C, and CX3CR1 were used to distinguish resident macrophages ($CD45^+/Ly6C^-/CX3CR1^+$), infiltrating monocytes ($CD45^+/Ly6C^+/CX3CR1^-$), and infiltrated monocyte-derived macrophages ($CD45^+/Ly6C^+/CX3CR1^+$); $n = 3$–5 POI mice per group.
E Representative confocal images of GFAP (violet)-positive EGCs and CX3CR1-GFP-positive macrophages (green, white arrows) around ganglia of intestinally manipulated mice treated with ambroxol or vehicle (24 h after IM). Scale bar 50 μm.

Data information: In (B), data are represented as IL-6 or CXCL2 protein in 100 μg tissue + SEM; $n = 6$ POI mice. In (C), data are represented as fold change + SEM. Statistics were done in (B and C) by applying unpaired Student's *t*-test and one-way ANOVA with a subsequent Bonferroni test. * indicates significance to sham animals, and $^{\#}$indicates significance between vehicle and ambroxol treatment with $^{\#}P < 0.05$, $^{**/\#\#}P < 0.01$, and $^{***}P < 0.001$.
Source data are available online for this figure.

 © 2021 The Authors

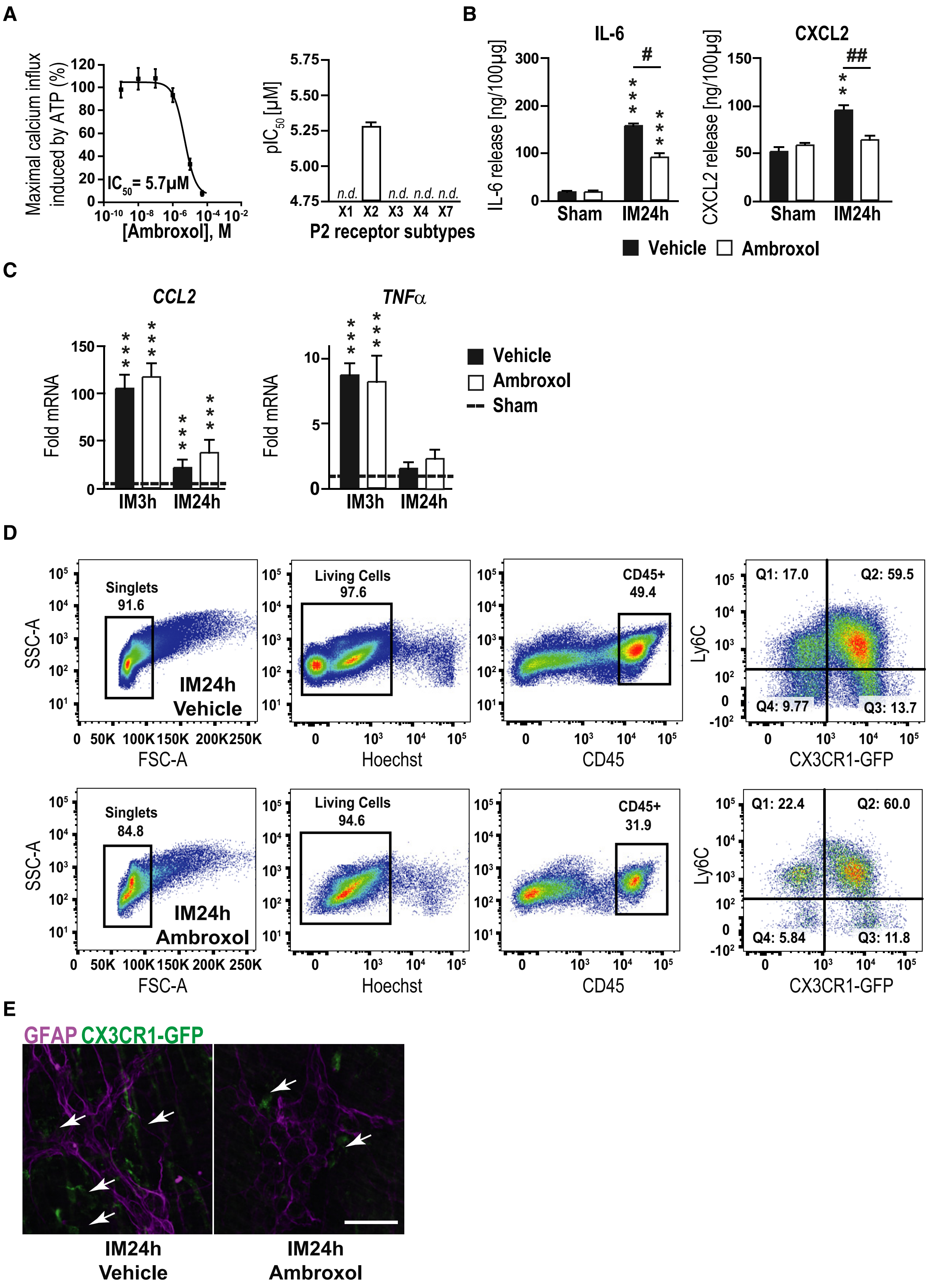
A
Maximal calcium influx induced by ATP (%)
IC_{50} = 5.7μM
[Ambroxol], M
pIC_{50} [μM]
n.d.
X1 X2 X3 X4 X7
P2 receptor subtypes
B
IL-6
IL-6 release [ng/100μg]
CXCL2
CXCL2 release [ng/100μg]
Sham IM24h
Vehicle Ambroxol
C
CCL2
TNFα
Fold mRNA
IM3h IM24h
Vehicle
Ambroxol
Sham
D
Singlets 91.6
IM24h Vehicle
Living Cells 97.6
CD45+ 49.4
Q1: 17.0
Q2: 59.5
Q4: 9.77
Q3: 13.7
Singlets 84.8
IM24h Ambroxol
Living Cells 94.6
CD45+ 31.9
Q1: 22.4
Q2: 60.0
Q4: 5.84
Q3: 11.8
SSC-A
FSC-A
Hoechst
CD45
Ly6C
CX3CR1-GFP
E
GFAP CX3CR1-GFP
IM24h Vehicle
IM24h Ambroxol

Figure EV4.

Figure EV5. ExATP induces gliosis in human enteric glia.

A PCA plot of gene expression from patient specimens at two different time points of the surgery; $n = 3$ for early and late specimens.
B IL-6 protein measurement in human surgical specimens collected during a pancreaticoduodenectomy at an early and a late time point of surgery. Samples were provided on ice directly from the operation room, and *muscularis externa* (ME) was separated from the lamina propria mucosae; $n = 9$ human patients.
C Gene expression analyses of gliosis marker in human surgical specimens collected during a pancreaticoduodenectomy at an early and a late time point of surgery. The late specimens' mRNA level show an up-regulation of gliosis genes.
D Immunofluorescence microscopy revealed P2X2 expression (green) in a majority of s100β^+ (violet) hEGCs in intact myenteric ganglia of the human colon. White arrows mark double-positive cells. Scale bar 50 μm.
E Immunofluorescence microscopy revealed P2X2 expression (green) in a majority of s100β^+ (violet) hEGCs in culture. DAPI counterstained nuclei. Quantification of double-positive cells showed that 75% of cultured hEGCs express P2X2 (marked with +). Scale bar 50 μm.
F Schematic workflow on the collection and processing of surgical specimens collected during a pancreaticoduodenectomy. Samples were provided directly from the operation room in oxygenated Krebs–Henseleit buffer and were mechanically activated *ex vivo*. Immediately after activation, specimens were incubated for 3 h with or without ambroxol (20 μM). Finally, ME was isolated and further processed for qPCR analysis.

Data information: In (B), data are represented as IL-6 protein in 100 μg tissue; $n = 9$ human patients. In (C), data are represented as fold change; $n = 13$ human patients. In (E), data are represented as the percentage of P2X2$^+$/s100β^+ cells + SEM; $n = 16$, hEGCs. Statistics were done by applying unpaired Student's *t*-test in (B, C). * indicates significance to control with $^*P < 0.05$, $^{**}P < 0.01$, and $^{***}P < 0.001$.
Source data are available online for this figure.

© 2021 The Authors

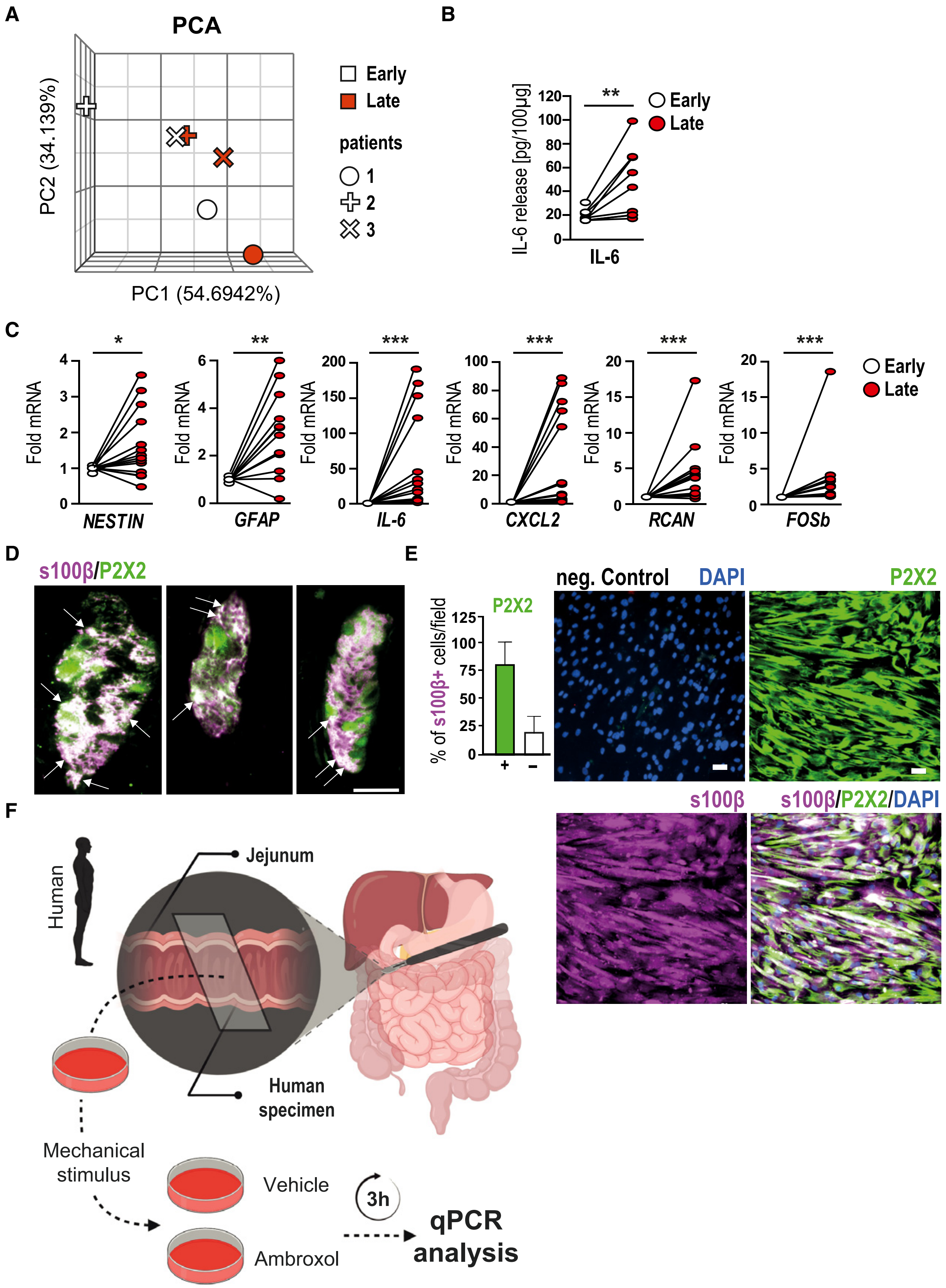
A
PCA
PC2 (34.139%)
PC1 (54.6942%)
Early
Late
patients
1
2
3
B
IL-6 release [pg/100µg]
**
Early
Late
IL-6
C
Fold mRNA
*
NESTIN
**
GFAP
***
IL-6
***
CXCL2
***
RCAN
***
FOSb
Early
Late
D
s100β/P2X2
E
% of s100β+ cells/field
P2X2
neg. Control
DAPI
P2X2
s100β
s100β/P2X2/DAPI
F
Human
Jejunum
Human specimen
Mechanical stimulus
Vehicle
Ambroxol
3h
qPCR analysis

Figure EV5.
